# Toposelective vapor deposition of hybrid and inorganic materials inside nanocavities by polymeric templating and vapor phase infiltration†

**DOI:** 10.1039/d2na00291d

**Published:** 2022-08-23

**Authors:** Ville A. Lovikka, Konsta Airola, Emily McGuinness, Chao Zhang, Marko Vehkamäki, Marianna Kemell, Mark Losego, Mikko Ritala, Markku Leskelä

**Affiliations:** Department of Chemistry, University of Helsinki A.I. Virtasen Aukio 1, P.O. Box 55 FI-00014 Helsinki Finland ville.lovikka@helsinki.fi cendel@gmail.com; School of Materials Science and Engineering, Georgia Institute of Technology Atlanta Georgia 30332 USA

## Abstract

Selective deposition of hybrid and inorganic materials inside nanostructures could enable major nanotechnological advances. However, inserting ready-made composites inside nanocavities may be difficult, and therefore, stepwise approaches are needed. In this paper, a poly(ethyl acrylate) template is grown selectively inside cavities *via* condensation-controlled toposelective vapor deposition, and the polymer is then hybridized by alumina, titania, or zinc oxide. The hybridization is carried out by infiltrating the polymer with a vapor-phase metalorganic precursor and water vapor either *via* a short-pulse (atomic layer deposition, ALD) or a long-pulse (vapor phase infiltration, VPI) sequence. When the polymer-MO_*x*_ hybrid material is calcined at 450 °C in air, an inorganic phase is left as the residue. Various suspected confinement effects are discussed. The infiltration of inorganic materials is reduced in deeper layers of the cavity-grown polymer and is dependent on the cavity geometry. The structure of the inorganic deposition after calcination varies from scattered particles and their aggregates to cavity-capping films or cavity-filling low-density porous deposition, and the inorganic deposition is often anisotropically cracked. A large part of the infiltration is achieved already during the short-pulse experiments with a commercial ALD reactor. Furthermore, the infiltrated polymer is more resistant to dissolution in acetone whereas the inorganic component can still be heavily affected by phosphoric acid.

## Abstract 2

The paper presents a nanoselective all-vapor workflow for depositing hybrid and inorganic materials inside nano- and micro-scaled cavities.

## Graphical abstract

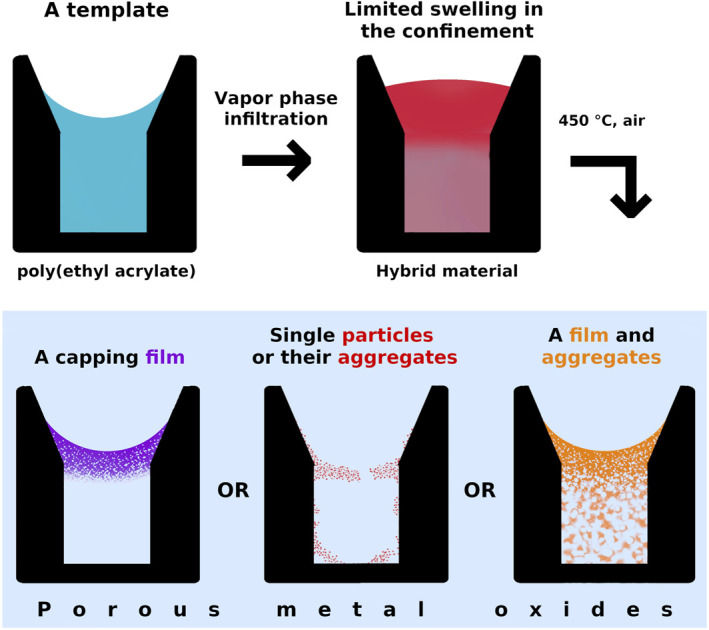

## Introduction

Efficient yet precise coating methods are necessities for modern nanotechnology. As the ambitions for future technologies grow, so do the requirements for technological advances. For example, in microelectronics, where new 3D chip infrastructures are needed to get beyond Moore's law, new kinds of selective coating and material converting methods are needed with nanoscale accuracy.^1,2^ An especially difficult task is to functionalize or fill nanocavities, and even more so, if the coating is to be location selective. Therefore, new approaches have been requested and sought after.^3–10^

Hybrid materials have gained interest for their properties that may exceed those of their components.^11,12^ Some applications, for example improved adhesives, require hybrid material to be located specifically in tight spaces between surfaces.^13^ However, a ready-made hybrid material is difficult to introduce inside cavities because it is typically stiff. A stepwise deposition can overcome this challenge, where a cavity-deposited matrix is converted into hybrid material. Furthermore, cavity-selective hybrid material can be calcined into cavity-selective inorganic nanoparticle deposition.

Having a workflow entirely in vapor phase would remove issues relating to solvents, *e.g.*, risks to the environmental and human health, surface contamination, low nanopore penetration, and immiscibility of the components. A vapor phase preparation of hybrid materials could be integrated into current industrial processes, yet the methodology is still in its infancy.^12^

Condensation-controlled toposelective vapor deposition (CTVD) is a method for cavity-targeted functionalization.^9^ First, a reagent is allowed to condense inside nanocavities, pores, interstices, ledges, and other places which have been previously very difficult to reach. This is possible *via* capillary condensation (CC), which arises from the vapor pressure difference over a curved gas–liquid surface, the meniscus, which stabilizes the liquid phase in small cavities if the contact angle is <90°.^14^ The to-be-filled cavity size can be selected by adjusting the partial pressure of the adsorbate. The condensate can be fixed in place into an area-selective deposition. Recently, we showed a self-built and affordable reactor setup that can be used for polymeric vapor deposition inside nanocavities in ambient conditions with photochemical fixation.^15^ CTVD has also been applied with setups ranging from commonplace glassware to commercial reactors.^9,16–18^

Vapor phase infiltration (VPI) is a chemical vapor deposition (CVD) method originating from atomic layer deposition (ALD) experiments carried on polymeric substrates.^19–22^ In ALD, a substrate is exposed to gaseous reagents in a subsequent fashion. The reagents react in a self-limiting manner with the substrate surface modified by the previous reagent pulse. In VPI, however, the reagents are not only adsorbed on the surface but absorbed inside the free volume of the polymer, making the growth volume-filling instead of surface-covering. While the film thickness in ALD is dependent on the number of cycles, the extent of VPI is dependent on lengths of reagent exposure and removal *via* purging due to the time-dependent diffusion, adsorption, and desorption of the precursors.^23,24^ When the pulsing length is increased from ∼1 s to minutes or even hours, and the number of cycles is reduced from thousands to single-digit numbers, inorganic material growth happens almost exclusively inside suitable polymer templates. The inorganic loading and chemical structure will also vary with precursor chemistry, polymer chemistry, and polymer structure.

In this way, the VPI process is more complex than ALD: the penetration depth, extent of reaction, reaction mechanism, covalent/coordination bonding, and many other factors are determined by the interactions between the polymer and the reagents, structure of the substrate, applied time, and reaction conditions.^20^ The reagents may simply get trapped inside the substrate and react with the next reagent pulse,^25^ or they might coordinate and react with the functional groups in the polymer.^26,27^ In either case, VPI offers a way to distribute inorganic material mixed at the atomic scale into the polymer matrix. Hybrid materials prepared with VPI are showing great promise for various applications: increasing etch resistivity of the polymer for nanopatterning,^28–31^ enhancement of mechanical or electrical properties as well as chemical and biological stability of the polymer,^32–37^ and many others.^20,38^ The material can be further processed into inorganic structures which replicate the original shape of the polymer template, which enables the fabrication of complex inorganic structures with sizes ranging from few nanometers to micrometers.^39–46^

In this paper, an all-vapor nanocavity-selective deposition of hybrid fillers and inorganic particles is demonstrated. First, cavity-grown poly(ethyl acrylate) templates were turned into hybrid materials with short exposures in a commercial ALD reactor, and long exposures in a custom-built VPI reactor. Previously, ALD and VPI have led to different degrees of infiltration inside non-constrained polymeric templates,^47^ but in this study, the difference was relatively small, which was partially attributed to higher temperature during the short-pulse protocol. Furthermore, the maximum infiltration was limited inside the most constrained cavities due to confinement effects, *e.g.*, reduced polymer chain mobility^48^ and limited space for swelling. The hybrid materials were further calcined into toposelective inorganic structures that were typically either cavity-filling porous deposition, cavity-bridging films with various degrees of anisotropic cracking, or particulate deposition inside the cavities. Possible causes for the differences in deposition are discussed.

## Methods and materials

### Condensation-controlled toposelective photoinitiated chemical vapor deposition (CT-piCVD)

Poly(ethyl acrylate) (pEA) was deposited inside nanocavities with a self-made CTVD reactor supplemented by a UV lamp.^15^ The coatings were made surface-selective by following the CTVD principles of controlling the atmosphere and delaying the initiation.^9^ The samples were held in a N_2_ atmosphere containing ethyl acrylate (>99%, TCI) at the saturation point for thorough capillary condensation on the surfaces. After at least one hour of exposure, the gas feeds were closed, and the low-pressure Hg lamp was turned on for 7–8 minutes to fix the condensed monomer into a polymer through UV-induced self-initiation.^49^ Some samples were prepared with 4% or 20% O_2_ content in N_2_ (synthetic air, 20% O_2_ in N_2_, Oy Aga Ab), and the initiation time was doubled to 15 minutes to counter the inhibitory effect of oxygen. The substrates were anodized aluminum oxide (AAO) with nominal diameters of 120–160 nm and depths of 0.5–1 μm (InRedox) and scratched internal surfaces of shattered Fisherbrand glass pipettes (Thermo Fisher Scientific). Caution is recommended with the monomers because heating acrylates increases the risk for a violent self-accelerating polymerization.^50^ Furthermore, deoxygenating incapacitates the inhibitor of the monomer.

### Vapor phase infiltration (VPI)

#### Short-pulse exposures with ALD reactors

The short-pulse infiltration experiments were done in an ALD reactor with AlO_*x*_ and TiO_*x*_ (R-150 reactor, Picosun), and ZnO_*x*_ (flow-type F-120 reactor, ASM Microchemistry). AlO_*x*_ was grown from trimethylaluminum (TMA) (Volatec Oy) and deionized water with N_2_ (99.999%, Oy AGA Ab) as carrier and purge gas. The AlO_*x*_ depositions were done for 10 cycles at deposition temperatures of 60, 85 and 120 °C. Each cycle consisted of a 6 s TMA pulse, followed by either a 5 or 30 s N_2_ purge. Water was then pulsed over 60 s with a 300 s N_2_ purge. The TiO_*x*_ and ZnO_*x*_ depositions were carried out at 120 °C using TiCl_4_ and diethyl zinc (DEZ) as the metal precursors, respectively. The deposition cycles were kept otherwise similar to that of AlO_*x*_, but only a 5 s N_2_ purge was used after the metal precursor dose. For the AlO_*x*_ process, having a much larger number of cycles would eventually create a poorly permeable metal oxide layer on top of the polymer,^51^ which, therefore, lead to ALD instead of VPI.^52^ Special caution is advised with TMA, because it is highly reactive and it ignites in contact with air. TMA pulses longer than 6 s were not considered due to acute risks to the reactor pump with the continuous-flow setup.

#### Long-pulse exposures with a VPI reactor

VPI was conducted in a custom-built reactor that supports static atmospheres.^53–55^ The substrates were purged with N_2_ for 2 hours before pumping the chamber to vacuum for 5 minutes. The samples were then exposed to a single pulse of metal reagent for 15 hours at 70 °C, purged and pumped for 5 + 5 minutes, and exposed to water vapor for 3 hours. There was no repeated cycling in these experiments in contrast to the ALD processes described above. The reaction parameters were chosen to ensure drying of the polymers before the reaction and to allow thorough infiltration of the polymer. The metal reagent was trimethyl aluminum (TMA) at 0.5–1.2 Torr, TiCl_4_ at 0.3–1.6 Torr, or diethyl zinc (DEZ) at 0.6–1.2 Torr as described in further detail in the ESI (Fig. S1†).

### Scanning electron microscopy (SEM) and energy dispersive X-ray spectrometry (EDS)

Polymer was removed from some of the SEM samples by heating them in air to 450 °C and holding the temperature for 3 hours. The samples were attached on aluminum stubs by carbon tape and then sputtered with a 1.5–5 nm layer of Au/Pd before imaging with a field emission SEM (Hitachi S-4800). AAO samples were pressed on the tape with a needle, which caused the surface to bend locally and create fresh cracks, which were then imaged for cross-sectional analysis. The EDS spectra were measured with 5 or 10 keV (Oxford INCA 350 connected with the FESEM). The AlO_*x*_ thicknesses were calculated from the *k* ratios of aluminum Kα X-ray lines using a GMRFilm program^56^ while presuming a density to be 3 g cm^−3^ and oxygen to be in a stoichiometric amount with aluminum. A FEI Quanta 3d 200i FIB-SEM likewise equipped with INCA 350 was used for top-down EDS mapping of VPI polymer regions on surfaces. Local Focused Ion Beam (FIB) erosion of the surface was done in order to gain insight on the ability of VPI to reach inside polymer within nanopores. For the FIB work, a gallium ion energy of 16 keV and side-angle milling geometry (52° from the surface normal) was used in order to minimize material mixing on the milled surface.

### Qualitative dissolution experiments

The stability of AlO_*x*_–pEA composite was tested against the dissolution of polymer or alumina in acetone or phosphoric acid, respectively. The poly(ethyl acrylate) on glass was infiltrated with the short-pulse AlO_*x*_ (120 °C, 30 s) program. The polymer dissolution test was carried in 50 ml of acetone (>99%, VWR) for 60 minutes while the solvent was stirred at 60 rpm with a magnetic stirrer. The acid dissolution experiments were done on samples that had been prepared at 85 °C or 120 °C with a 30 s purge after the TMA pulse. The samples were placed into 50 ml of 5% phosphoric acid solution for 10 minutes while the solution was being stirred at 60 rpm. This should cause only a mild etching of dense alumina, because a 5% solution etches alumina at a rate of <1 nm min^−1^ at 37 °C.^57^ On the other hand, dissolved aluminum is readily precipitated as phosphate (*K*_SP_[AlPO_4_] = 6.3 × 10^−19^).

## Results

**Fig. 1:**
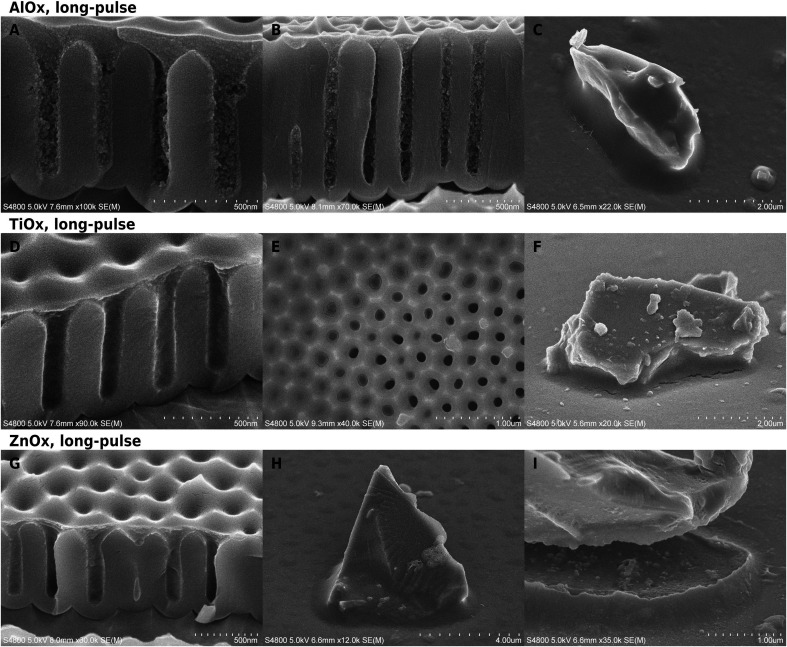
SEM images of long-pulse hybrid materials on glass and AAO. AlO_*x*_: cross-section images of AAO substrates (A and B) and a tilted view on (C) glass. TiO_*x*_: a tilted cross-section (D) and top-view (E) on AAO, and a tilted side-view on (F) glass. ZnO_*x*_: (G) cross-section of AAO and tilted side-views on glass with an intact (H) and broken (I) meniscus.

**Fig. 2:**
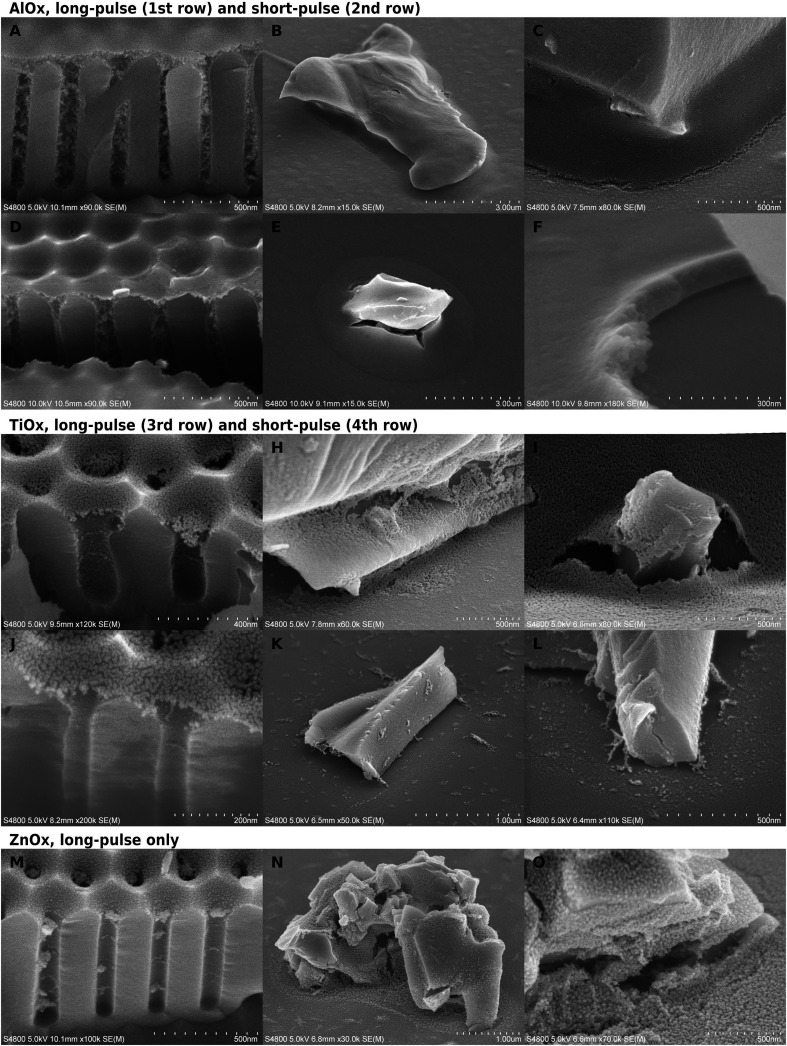
SEM micrographs of calcined VPI experiments on AAO (first column) and glass (second and third columns) substrates with either one cycle of long pulses or 10 cycles with short pulses. (A–C) Alumina with long pulses. (D–F) Alumina with short pulses. (G–I) Titania with long pulses. (J–L) Titania with short pulses. (M–O) Zinc oxide with long pulses.

**Fig. 3:**
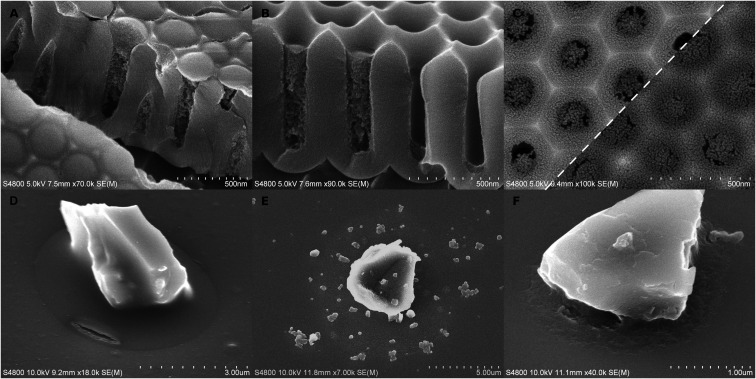
Further details on long-pulse VPI depositions (A–C) before or after calcination and dissolution experiments with short-pulse samples (D–F). (A) Swollen pEA in AAO pores after long-pulse AlO_*x*_ infiltration. (B) Alumina–pEA hybrid material filling pores to different degrees (C) A two-image composite comparing titania films on AAO after calcination. (D) Alumina–pEA hybrid material after 1 h of acetone dissolution. (E and F) Alumina after 10 min dissolution with 5% phosphoric acid with the polymer having been burnt either after (E) or before (F) the dissolution experiment.

To make reading of the SEM images easier, the reader is encouraged to inspect the image series showing AAO and glass samples at different stages of the workflow (Fig. S2 in the ESI†). Pure polymer is especially difficult to see in small amounts, and therefore careful examination is needed. The VPI process appeared well reproducible, however, the polymeric predeposition had variations mentioned later in the text. The results are first presented for hybrid materials (Fig. 1), then inorganic materials (Fig. 2 and Table 1) and further elaborated with Fig. 3 and S3–S6.† Unless otherwise noted, the short-pulse infiltration experiments were carried at 120 °C.

**Table 1:** Qualitative characteristics of inorganic depositions after the calcination. In this table, films well above 10 nm in thickness are considered “thick” irrespective of their porosity

		AlO_*x*_		TiO_*x*_		ZnO_*x*_	
		AAO	Glass	AAO	Glass	AAO	Glass
VPI (long pulse, 70 °C)	Meniscus	Thin film if in the cylindrical pore part, thick and denser if above	Thick film	Thin film	Thin film but thicker than on AAO	Particle aggregates	Collapsed granular film or particle aggregates
	Below the meniscus	Flake-like aggregates	—	Thin ring-like depositions	Empty or single particles	Particle aggregates	Single particles or their aggregates
ALD (short pulse, 120 °C)	Meniscus	Thin film if in the cylindrical pore part, thick and denser if above	Thick film	Thin film	No film, collapsed remains	No film	No film
	Below the meniscus	Flake-like aggregates	—	Thin ring-like depositions	Separate particles or ribbon-like aggregates	Empty	Empty or little deposition

### Appearance of the hybrid materials

The pEA templates appeared thoroughly infiltrated in all SEM cross-section images of non-calcined samples except for the short-pulse ZnO_*x*_ experiments. The hybrid material does not behave like the original pEA: it is easier to image due to higher electron-stopping power, it does not flow upon electron bombardment or cracking of the substrate surface, and the cross-section has texture instead of being hard-to-observe featureless mass.

Structural changes upon infiltration were evident in cross-section images (Fig. 1). The cross-sections varied from smooth to clumpy or slightly jagged, depending on the location and the chemistry. The largest changes were in long-pulse alumina infiltration experiments at temperatures above 60 °C, which created dense textured material on AAO pore mouths (Fig. 1A and B). Deeper inside the structures, where the conical part of the pore mouth changed into straight-walled cylinders, the material appearance became clumpy. The AlO_*x*_ had infiltrated well even pores with a higher aspect ratio (Fig. 1B) where a slight gradient in the deposition is visible along the cylindrical part of the pore. Short-pulse AlO_*x*_ infiltration at 60 °C was less thorough and its appearance was closer to that of titania infiltrated samples than the other alumina experiments (Fig. S5†). TiO_*x*_ (Fig. 1D–F) and ZnO_*x*_ (Fig. 1G–I) did not cause as clear textural changes as AlO_*x*_ deposition. The pore-filling material had jagged cross-sections and it was still more easily distinguishable in SEM than pure polymer fillers. A very thin surface film was easy to see on the surface from cross-section images. There was little difference between short and long-pulse experiments in case of titania and alumina, but the short-pulse ZnO_*x*_-VPI (120 °C) left the polymer seemingly uninfiltrated while the long-pulse experiments (70 °C) caused clear hybridization.

Cracking of the hybrid material was rare or very small in long-pulse experiments. Glass particles had sometimes moved in the experiments which had caused menisci to crack open (Fig. 1I) instead of accommodating by flowing. The meniscus shape of the alumina–pEA hybrid material was reversed under specific conditions. If the pores were filled up to the pore mouth without overfilling, the concave meniscus was reversed into a convex bulge (Fig. 3A). However, if the porous surface of AAO was overgrown by the polymer, small concave recessions remained on top of the pores, or if the meniscus was located deeper in the AAO pore (Fig. 3B), no reversing occurred.

The amount of metal followed closely the amount of pEA carbon (Fig. S3†) in the top-down EDS elemental maps of titania and zinc oxide. There was more metal deposited in the areas where the polymer has grown, but there was also a weak metal signal from all areas and a faintly elevated metal signals in spot-like areas that were not properly visible in top-down SEM. Focused Ion Beam milling was applied in order to investigate the extent of metal incorporation deeper in the pores. An ion dose of 1.28 × 10^17^ ions per cm^2^ (nominal milling depth 50 nm) was used to remove the surface layer, and a dose of 2.56 × 10^17^ ions per cm^2^ (100 nm) to mill deeper into the porous material (Fig. S4†). The Ga elemental map confirms that the milling was well contained within the intended locations. In titania samples, both C and Ti signals dropped notably with the 1.28 × 10^17^ ions per cm^2^ dose. Ti signal dropped slightly faster than C signal after the first milling dose, however, the difference was not large. Milling with twice the dose did not bring notable further change, which might be because the first milling already reached the cylindrical pore part or it removed the potentially signal-amplifying Au/Pd protective layer.

### Allocation and quality of inorganic deposition after calcination

When the polymer was burned away, an inorganic phase remained in varying thicknesses and forms. The calcination left a cavity-bridging film, aggregates, a field of scattered particles in the cavities, or a combination of these. The most extensive infiltration was in the long-pulse alumina experiments (Fig. 2A–C), which remained as thick self-standing films replicating the typical polymer surface. There was usually a gradient of less densely grown inorganic deposition below the thick surface layer. The most scarce depositions were with short-pulse zinc oxide with no observed infiltration, and short-pulse TiO_*x*_ that took the form of either a very thin film on the pore mouths (Fig. 2J) or particulate clutter inside the cavities (Fig. 2K and L).

The temperature had a notable effect on short-pulse AlO_*x*_ deposition thicknesses, as can be expected due to the temperature dependent diffusion of the precursors and the dimerization of TMA at low temperatures.^53,58^ When the temperature was lowered to 60 °C, the surface film inside cavities became thinner and the cylindrical part of AAO pores had less aggregates and sometimes only belts, a bit reminiscent to the titania depositions (Fig. S5†). There was good alumina infiltration only when the polymer had overgrown the AAO pore mouths. However, there was little difference in the infiltration between 85 and 120 °C in the short-pulse experiments even according to the EDS analysis. The films grown at 60 °C were much thinner than those grown at higher temperatures also on the glass substrate.

Both infiltration protocols deposited more material than an ALD process would deposit on a dense flat surface. According to the thickness analysis by EDS, a short-pulse alumina film on glass corresponded to a dense 40–45 nm alumina layer. This is almost 40-fold in thickness to a dense 1.2 nm ALD film grown over 10 cycles. In SEM images, the infiltrated film thickness appeared about twice as thick as indicated by EDS, which suggests that half of the film volume was void (Fig. 2F). However, the lateral and depth resolution in EDS is not good enough for having a good quantitative reading from nanostructured substrates. The long-pulse experiments were expected to bring a massive increase in the deposition in comparison to short-pulse infiltration, because the deposition time was larger by orders of magnitude. However, the long and short-pulse experiments had rather comparable depositions except for the short-pulse alumina at 60 °C and short-pulse ZnO_*x*_.

TiO_*x*_ depositions were much thinner, only *ca.* 10 nm on AAO (Fig. 2G and J), than those of AlO_*x*_ (Fig. 2A and D), and the pores appeared empty besides some belt-like formations. On glass, the long-pulse TiO_*x*_ VPI (Fig. 2H and I) gave a slightly thicker meniscus-shaped film than on AAO, whereas short-pulse experiments on glass (Fig. 2K and L) resulted only in separate particles and scarce cavity-bridging ribbon-like aggregates (Table 1). Scattered particles were located in front of the aggregates, which suggested that the aggregates can not be located where the polymer surface had originally been. This is different from all other depositions, because typically a significant portion of the deposition was located as a film in place of the uncovered template surface.

In the long-pulse experiments, ZnO_*x*_ deposited more than TiO_*x*_ but less than AlO_*x*_ (Fig. 2M–O). ZnO_*x*_ was aggregated inside the AAO, but the aggregates did not form as continuous as with alumina (Fig. 2M). The TiO_*x*_ and ZnO_*x*_ films looked often as if they had been “deflated”: films were partially resting on the substrate with edges laying flat on the surrounding surfaces. The effect was the most obvious on glass samples of long-pulse TiO_*x*_ (Fig. 2I) and the granular ZnO_*x*_ (Fig. 2N), where the films had to span greater distances than on AAO. In comparison, alumina films looked relatively unchanged from the shape of the template surfaces.

Besides the reagent chemistries, VPI was also restricted by the cavities. One correlating factor seemed to be the cavity geometry. As a thumb rule, the more constrained the space was, the less deposition was left of the hybrid material after calcination. For instance, a pore in AAO has two parts, a short conical part, and below it, a cylindrical part. VPI was slightly limited already in the conical part in comparison to VPI on glass (Fig. 2). For titania, the film appeared thinner and less intact if it was located more towards the bottom than the top of the conical part (Fig. 3C). The deposition was the weakest (Fig. 2A, D and M) and, for titania, almost non-imageable (Fig. 2G and J) inside the cylindrical part of the pores.

Even when there was only little inorganic deposition, faint ring-like structures were formed in the pore walls after long (Fig. 2G and M) and short-pulse (Fig. 2J) processes. The most open cavities were on glass substrates, where even solid wall-like depositions were left in place of the original template. If the deposition was very granular or it did not form self-standing structures, walls (Fig. 2O) or cavity-bridging ribbon-like depositions (Fig. 2K and L) were formed deeper in the cavity than where the polymer surface had likely been. Interestingly, while there were varying amounts of particulate-like deposition in front of these depositions, the deposition was much more scarce deeper in the cavities (Fig. 2F, H, I, K and L). In general, the tightest cavity corners looked relatively clean even when there was deposition in their surroundings in both short and long-pulse experiments.

Some infiltration experiments were repeated on pEA that had been initiated in an O_2_-containing atmosphere for 15 minutes (Fig. S6†). Oxygen increases CTVD selectivity by reducing the polymeric deposition on open surfaces, but then a prolonged initiation is needed to counter the inhibitory effect inside the condensed monomer. These two changes might affect the polymer structure because it becomes clumpier.^15^ The deposited inorganic film appeared thinner after calcination, and it had often more clear-cut edges than if only N_2_ was used as the carrier gas.

The inorganic film was often cracked depending on the substrate and the process. Generally, the most intact films were produced by the long-pulse AlO_*x*_ sequence. The location of the crack varied between the middle of the meniscus and the contact point with the substrate, but it typically followed the contours of the substrate surfaces. This tangential cracking was occasionally accompanied by smaller radial cracks (Fig. 2E). The cracking was dependent on the location of the meniscus. TiO_*x*_ films had less cracking if the meniscus was located higher in the conical pore opening (Fig. 3C). The cracking in alumina-infiltrated samples was more extensive around glass particles than in AAO pores (Fig. 2A–F).

### Dissolution experiments

Practical implications of VPI were experimented with short-pulse alumina samples. VPI enhanced the resistance of cavity-grown poly(ethyl acrylate) against dissolution. The polymer without VPI was dissolved into acetone within a minute. Instead, short-pulse treated samples retained 50–80% of the polymer even after 60 minutes of dissolution. The result was determined by EDS measurements by comparing the Al/C ratio with acetone treated and as-deposited samples. Furthermore, the hybrid material was occasionally cracked (Fig. 3D), resembling the depositions of alumina after the polymer had been removed by heating.

The polymer did not prevent the dissolution of AlO_*x*_. The hybrid material appeared unchanged right after the phosphoric acid treatment, but after calcination, particles remained instead of a film (Fig. 3E). This effect occurred already with 1% phosphoric acid in 1 minute. It is undefined whether the particles formed during calcination or if they had space to form already inside the non-crosslinked poly(ethyl acrylate). Surprisingly, if the polymer was burned away before the acid etching, the remaining inorganic phase was more continuous and retained a damaged yet recognizable meniscus shape encircling the contact point between the particle and the substrate floor (Fig. 3F).

## Discussion

The difference in inorganic material distribution before and after calcination was striking especially with titania samples. From cross-section images of the hybrid materials it is clear, that some titanium did reach deeper parts of the pores during VPI, yet after calcination, there was apparent loss of almost any inorganic filler inside the cavities. The observations before and after calcination do agree though, that there is a thin zone of stronger infiltration on the free surface of the polymeric template.

The Ti (and Zn) signals correlated well to C signal (Fig. S3†) in EDS top-down mapping across the AAO surfaces. The difference between Ti/C ratio in non-milled and milled surfaces is not as large as what would be expected based on SEM images of calcined samples. Some metal was present on all areas but the amount was much smaller in the empty-looking than in the polymer-infiltrated areas. The background signal was probably from hybrid materials in hard-to-observe underfilled pores and ALD growth on polymer-free surfaces. Even that a small portion of EDS signal comes from the surfacemost 10 nm, and therefore, EDS downplays the significance of very thin surface film, there should be infiltrated titania also inside the cavities.

The formation of thin TiO_*x*_ films instead of more continuous deposition might be related to the higher mobility of the free-surface polymer chains,^59^ which could then accommodate absorption much more easily than the bulk below. TiCl_4_ is known to saturate surfaces rapidly and cross-bridge pMMA in comparable conditions.^60^ However, the infiltration of TiO_*x*_ did not stop to the surface layers although it appears weaker, and long-pulse one-cycle exposure did not improve infiltration notably. The calcination step might have reallocated the deposition inside the pores: titania may have been pulled deeper in the cavity during the polymer removal, which would enrich the material on the pore walls, where occasionally belts (Fig. 2G and J) or cavity-bridging ribbons (Fig. 2K and L) were formed.

Also alumina and zinc oxide were redistributed during calcination, but the amount of material was so large that all of it could not retract to the pore walls. The deposition stayed in place or formed easily observable aggregates. A large portion of zinc oxide particles were jammed together in aggregates or irregular films (Fig. 2M–O). Alumina created very thick films near the free surface of the polymeric templates, and substrate-supported flake-like aggregates deeper in the cavities. However, with the short-pulse experiments at 60 °C, alumina yield was lowered and the results reminded those of titania. The infiltration during TMA–H_2_O process seems to be strongly temperature dependent. Although TMA is almost exclusively dimer at such low temperatures,^58^ alumina process has lead to manyfold thicker coatings at 70 °C than 130 °C.^53^ Nevertheless, in our experiments, the low infiltration during the short-pulse experiment at 60 °C was striking in comparison to the long-pulse experiment at 70 °C or short-pulse experiment at 85 °C.

The strong infiltration of alumina is supported by favorable interactions between TMA and the carbonyl groups^26^ of pEA. Strong interactions help with diffusion but may lead to chemical reactions and cross-linking at temperatures above 100 °C, which could hinder further infiltration.^27^ The infiltration is supported also by the low glass transition temperature (*T*_g_) of pEA (*ca.* −11 °C for atactic pEA,^61^ however, constrainment affects even *T*_g_,^62^ and polymers in AAO nanopores or concave surfaces may face *T*_g_ changes in scale of 10^1^ °C (ref. 63 and 64)), because pEA would be viscous instead of brittle in the infiltration temperatures due to increased chain mobility.^65,66^ Therefore, poly(ethyl acrylate) is expected to have plenty of free volume that helps with the uptake of reagents.

Poly(methyl methacrylate) (pMMA) serves as an interesting comparison point in lack of VPI literature on pEA. Although pMAA has considerably higher *T*_g_ (*ca.* 100 °C (ref. 67)), it is structurally very close to pEA. Most of our alumina infiltration was reached in the time scale of the short-pulse experiments. This is close to the results from TMA infiltration experiments of pMMA on open surfaces, where the swelling plateaued after 10 s at 130 °C or 6 h at 70 °C.^53^ Our short-pulse experiments were carried at higher temperature (mostly 120 °C) where the infiltration is more rapid meanwhile the long-pulse experiments (70 °C) are expected to be more extensive. However, nanocavities would be problematic to the degrees of swelling that are related to such infiltration. TMA–H_2_O pulse on pMMA could reach dozens of nanometers deep in 10 s and cause full-cycle swelling by over 15% or 40% at 130 °C or 70 °C, respectively.^53^ Maximum swelling during the process had been even higher, because the material deswells during the H_2_O pulse. Another paper reported that a TMA–H_2_O cycle with a very long TMA exposure was used to convert 60 nm thick pMMA film to 19 nm alumina film at 90 °C.^46^ Confusingly, part of the swelling might result from increasing free volume instead of material addition: a burnout of AlO_*x*_–pMMA left an inorganic film that was only 70–80% of the excess volume caused by swelling during VPI at 70–130 °C.^32^

Drawing reliable conclusions on why the infiltration was different with different precursors is difficult. We are not aware of papers that would report diffusivities and solubilities of all our metal precursors inside similarly prepared acrylate films, but the literature reports similar results to ours. TMA + H_2_O has been more strongly filling than TiCl_4_ + H_2_O on pMMA as well.^22^ TiCl_4_ molecule has a similar size to TMA, but TiCl_4_ has a more round shape which may be disadvantageous to its diffusivity.^20^ Deposition of ZnO_*x*_ inside pMMA has been more difficult compared to alumina, and typically a TMA + H_2_O cycle has been used to assist in nucleation before ZnO_*x*_ deposition.^68,69^

Interestingly, ZnO_*x*_ deposited well with long-pulse experiments at 70 °C but did not grow properly during short pulsing at 120 °C. Since diffusion and reactivity are better at higher temperatures, the reason is likely because of worse precursor detention: the temperature was still too low for reactions between DEZ and pEA, but so high, that the precursors would have desorbed before the water pulse.^70^ The amount of titania deposition was also very low considering that TiO_*x*_ could nucleate through an 180 nm thick pMMA film on flat surfaces during 2 second pulses at 160 °C.^71^

The infiltration of our samples varies depending also on the geometry, *i.e.*, the openness and width of the cavities. VPI should reduce gradually through a polymer film.^71^ Instead, on AAO, a change in deposition occurred where the pore mouth transitioned from conical to cylindrical geometry. The geometric effect is most notable in the most infiltrating process, alumina. The alumina deposition grew denser above cylindrical part irrespectively of how thick the layer was: if the polymer had grown over the pore mouths, the densest deposition was thicker by the corresponding amount (Fig. 2A and 3A, B). Even the TiO_*x*_ films appeared more intact and perhaps slightly thicker when the polymer surface had located at the upper part of the conical pore mouth (Fig. 3C). The differences between the cavities of AAO and glass were also clear albeit complex. Alumina films on glass substrates looked always denser than the deposition inside cylindrical part of an AAO pore. Titania films were somewhat thicker on glass but only in the long-pulse experiments. Short-pulse titania films did not survive calcination on glass, probably because they were too thin and the supporting substrate walls were far apart.

These geometrical dependencies could be connected to several different factors. One of the most certain factor is the constrainment effect caused by a nearby surface. A polymer deposited on a single flat surface may experience confinement effects up to dozens of nanometers deep. The polymer chains would lose mobility and appropriately sized fraction of free volume elements for adsorption.^59,72,73^ Constrainment would be even stronger in nanocavities, because the polymer is intimately contacted from multiple directions. Therefore, cavities would also restrict infiltration more than flat surfaces. This is supported by our observation, that the sharpest corners and the bottoms of the pores were often the most clean of detectable inorganic deposition.

VPI has been usually done to polymers on flat surfaces, where the material is free to swell upwards or, on some occasions, sideways by buckling.^74^ Phase-separated copolymer depositions have one more option, because non-swelling phases could accommodate expansion from adjacent swelling phases.^51^ In all cases, volume-creating phenomena are needed to increase the free volume inside the polymer and to allow VPI to continue. Despite of these difficulties in comparison and prediction of swelling, it could be argued, that even small amount of swelling can be troublesome when the constrainment is strong.

If the swelling is extensive as in the alumina experiments, the original concave “meniscus” can be flipped into a convex surface when it is hybridized (Fig. 3A). This is especially noteworthy considering that the swelling of a bent surface would probably exert forces towards the convex side and therefore oppose such meniscus flipping. The flipping appeared much smaller or non-existent if the hybrid material surface was located deeper in the pores (Fig. 3B). If the meniscus was located near the bottom, even the alumina-infiltrated polymer remained concave. There are various possible explanations: stronger confinement might limit the diffusion and swelling more than the conical part, or the cylindrical pore shape could support the original meniscus shape better than the conical part. A larger volume/surface ratio might also be needed before the swelling accumulates into larger shifts and flipping of the surface. However, this is in contradiction with a peculiar observation. The concave-convex flipping did not happen if the polymer had overgrown into a continuous film over the AAO surface (Fig. S2E†). This is counterintuitive, because the deposition is thicker in the pores than above AAO ridges by orders of magnitude, and there would be less constrainment effects above a pore, and therefore, the swelling should have been much more extensive directly above the pores. One possible explanation is that the swelling stress could be redistributed under a continuous surface that may have little adherence with the substrate.

Geometry of the pore may affect infiltration in many other ways. The films were thicker and denser on glass because the constrainment is less strong in a toroid deposition with an exposed outer edge (tapered confinement below a saddle-shaped free surface of the meniscus) instead of the AAO pores, where the polymer forms a straight-walled cylindrical plug with only one exposed end. Furthermore, there is less constrainment from glass, because the surfaces are usually further apart than in cylindrical AAO pores. The ratio of polymer volume to its free surface is smaller on glass than AAO, and therefore, similar amount of infiltration would cause smaller shifts in surface positions on glass than AAO. Besides, the diffusion is easier on the glass substrate and the conical part than in cylindrical part of the AAO pores, because the cavities have more open shapes allowing a larger variety of diffusion pathways.

Interestingly, polymers that were deposited with added O_2_ in the atmosphere resulted in inorganic films that appeared slightly more controlled in their area-selectivities and thicknesses. Fidelity in the XY plane is probably caused by the better-defined deposition of the polymer template, whereas the more precise film thickness could be from altered polymer structure.^15,75^ Reactive oxygen species can modify even polymers without proper functional groups. For example, polypropylene gained polar side groups and cross-bridges when it was irradiated with UV under an oxygen-containing atmosphere, and similar treatment has been used to enhance VPI with polystyrene.^75,76^ New functional groups would increase reactions in the surface layers, which, in combination with a possible cross-bridging even without VPI precursors, might inhibit infiltration deeper in the polymer.

Polymer dissolution was reduced strongly by the VPI treatments despite the limited infiltration. Acetone dissolution caused a large fraction of the polymer to be lost, but the composite shape was fairly well preserved (Fig. 3D). Our protective layer was much thinner than in the previously reported results, where a single AlO_*x*_ cycle on open surfaces was enough to prevent the dissolution of pMMA against strong solvents.^32^ Based on this, the capabilities of VPI to protect the polymers from dissolution might be reduced albeit not lost under constrainment. On the contrary note, the alumina was poorly protected against dissolution, because a scattered field of large particles, possibly aluminum phosphate, was formed. However, if the polymer was burned away before etching, the infiltrated alumina partially retained its meniscus shape. This is against the intuitive thought that the polymer would reduce the accessibility of inorganic deposition, and therefore, reduce its dissolvation.

The high temperature during calcination might cause sintering. Sintering would increase the particle size and reduce porosity which would make dissolution and further shape-altering effects, for instance, solvent-based sintering^77^ much slower. Although alumina has a very high melting point, the melting point depression of sub-nanometric particles might be enough to bring the threshold below 450 °C.^78^ Such sub-nanometric particles would be expected because VPI proceeds evenly throughout the polymer, which leads to very fine particles.^69^ Those particles would ideally grow into a continuous film only later as more VPI pulses are carried.^52^ However, sintering would explain the film stability only partially, because even alumina films remained rather porous in this study.

Cracking of the inorganic deposition might be related to factors that have not been present in the prior VPI literature, where the templates have been either self-standing or merely supported on a flat surface. For example, during calcination, the polymer is expected to burn starting from the surface, because oxygen is supplied from the interface with the atmosphere. The polymer surface is therefore retracted towards the bottoms of the cavities. The retraction could drag inorganic deposition along, which would cause bending and stretching in the film that is partially already connected to the substrate, pull already formed gaps wider through tensile forces, and cause particle accumulation and aggregation deeper in the cavities. The surface features of the substrate prevent isotropic shrinkage and contribute to higher stresses in more constrained cavities. Tensile stresses due to contraction inside cavities have been conceptualized in restorative dentistry as the *C*-factor, which is the ratio between bonded and unbonded surfaces between the filler and the cavity. The stress inside the cavity becomes larger as the *C*-factor is increased and shrinkage is more restricted, which risks the integrity of the filler in the cavity.^79^ The amount of stress is related to the restriction of polymer shrinkage if the filler can not flow.^80^

Cracking of hybrid material could happen already at room temperature when the polymer was partially removed by acetone dissolution, a process, which is also likely to start from the surface (Fig. 3D). There may also be stresses which were developed due to sintering, or during the cooling of the system, because the thermal stresses from differences in thermal expansion coefficients may be orders of magnitude larger than stresses that occur because of structural densification.^81^ However, the bending of the films suggests that the cracking is not solely from thermal contraction which ideally causes isotropic changes in size.

Understanding nanoscaled VPI requires studying the effects of differently shaped interfaces and cavities. The results would be interesting in various ways. They might help refine the novel reaction-diffusion transport model of VPI, where the swelling was neglected for simplification.^82^ There is rising interest in both organic and inorganic deposition methods on the liquid–vapor interface and creating canopies over nanostructures.^83^ Hybrid products with controlled dimensions could be of a high value.^20^ CTVD and VPI can be utilized to create those materials inside and above cavities. Unfortunately, the lack of suitable analysis methods for stochastic small-nuclei phenomena on large non-uniform surfaces is an acknowledged problem that hinders designing new nanoscaled vapor deposition methods.^10^ Important future tasks include finding methods to increase the total volume of infiltration in nanoconstrained templates, control the final particle distribution inside cavities, and create suitable measurement techniques and analysis tools for such depositions.

## Conclusions

The substrate geometry around the polymer templates was identified as a new factor affecting vapor phase infiltration. The pEA was hybridized in all cavities but less extensively than on open surfaces. The infiltration was the least limited by the confinement effects near the free polymer surfaces and in the most open substrate geometries. Polymer removal caused the inorganic material to reallocate and aggregate: different combinations of cavity-capping films and porous pore fillings, aggregates, and single particles were found instead of a gradually decreasing porous filler. Continuous inorganic depositions were often cracked, which might be a result of swelling-induced mechanical transformations, thermal expansion and shrinkage, anisotropically progressing sintering, or tensions from the anisotropic substrate during polymer removal and sintering. Even the limited VPI was enough to strongly reduce polymer dissolution from the hybrid material into acetone. However, the inorganic component could be easily transformed by dissolution in acid.

Vapor phase infiltration to cavity-grown polymeric templates is a promising all-vapor workflow to deposit hybrid and inorganic materials selectively in nano- and microcavities. A large portion of the maximum infiltration was reached already within tens of seconds in cavities when an appropriate temperature was used. The swelling behavior of hybrid materials can be complex inside small cavities, and it needs to be taken into account in understanding and modeling VPI in cavities, or when more extensive or better controlled infiltration is needed in practice.

## Author contributions

V. L. conceptualized and administered the study, co-designed infiltration experiments, prepared polymerized samples, analyzed the results, wrote the initial manuscript, and supervised K. A.; K. A. prepared polymerized samples, co-designed short pulse infiltration experiments, designed and executed the dissolution experiments, and helped with the manuscript; E. M. designed and ran the long-pulse infiltration experiments; C. Z. co-designed and ran short-pulse experiments; M. V. designed and executed elemental mapping of long-pulse experiments; M. K. analyzed the layer thicknesses, and supervised SEM operations and EDS experiments on short-pulse samples; professors M. Losego, M. R. and M. Leskelä supervised the study and provided feedback on the manuscript.

## Conflicts of interest

There are no conflicts to declare.

## Supplementary Material

NA-004-D2NA00291D-s001
