# Supplementary material for: Toposelective vapor deposition of hybrid and inorganic materials inside nanocavities by polymeric templating and vapor phase infiltration
Source: Nanoscale Adv. 2022 Aug 23;4(19):4102–13. doi: 10.1039/d2na00291d (PMC9514560; doi:10.1039/d2na00291d)
Supplement: NA-004-D2NA00291D-s001 [file NA-004-D2NA00291D-s001.pdf]

Electronic Supplementary Material (ESI) for Nanoscale Advances.
This journal is © The Royal Society of Chemistry 2022

Supporting info

Toposelective vapor deposition of hybrid and inorganic materials inside nanocavities by polymeric templating and vapor phase infiltration

Ville A. Lovikka[1,*], Konsta Airola[1], Emily McGuinness[2], Chao Zhang[1], Marko Vehkamäki[1], Marianna Kemell[1], Mark Losego[2], Mikko Ritala[1], Markku Leskelä[1]

*: Corresponding author: ville.lovikka@helsinki.fi, cendel@gmail.com
[1]: Department of Chemistry, University of Helsinki, A.I. Virtasen Aukio 1, P.O. Box 55, FI-00014 Helsinki, Finland
[2]: School of Materials Science and Engineering, Georgia Institute of Technology, Atlanta, Georgia 30332, United States

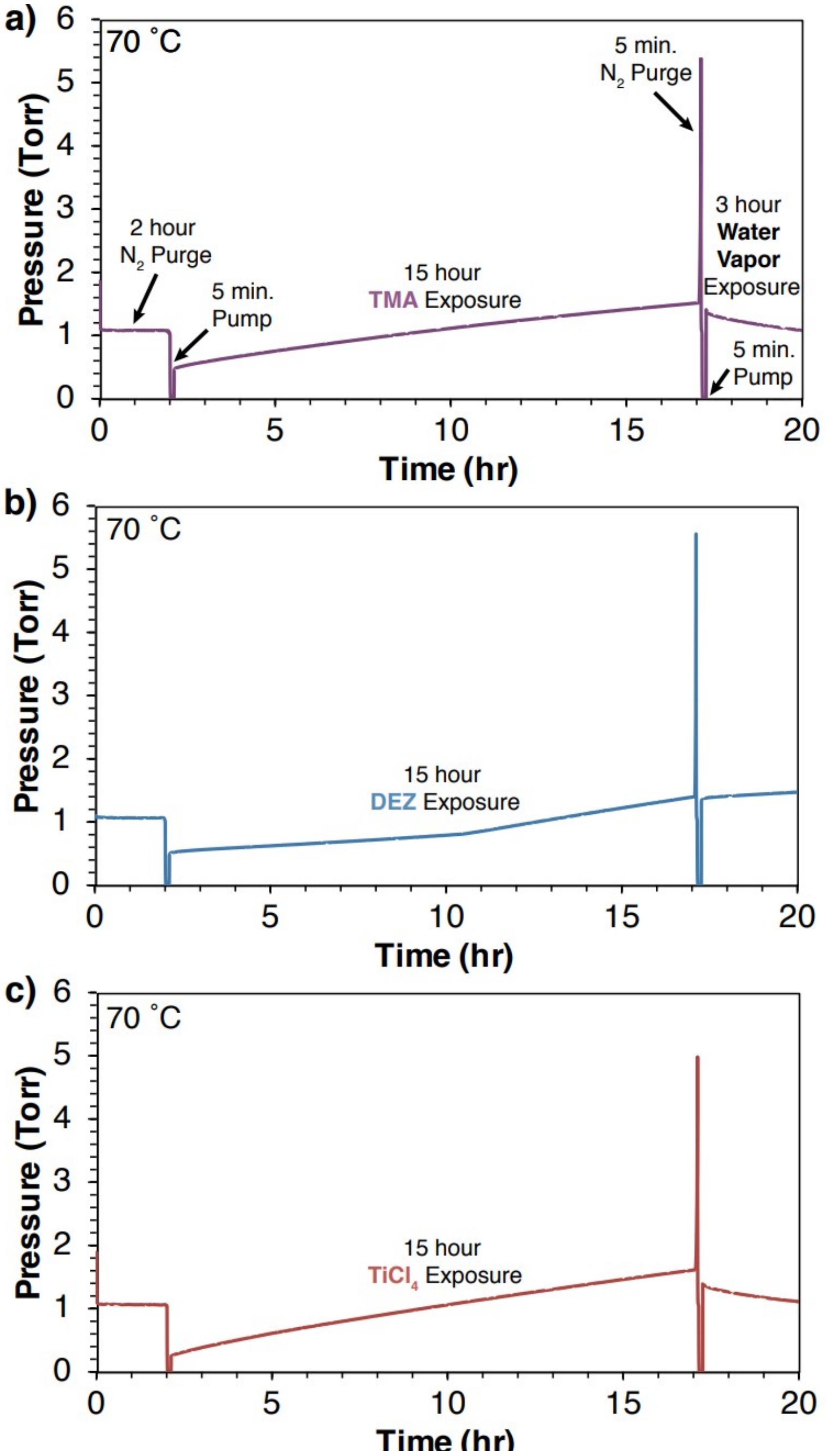

Figure S1: Experimental details about long-pulse VPI.

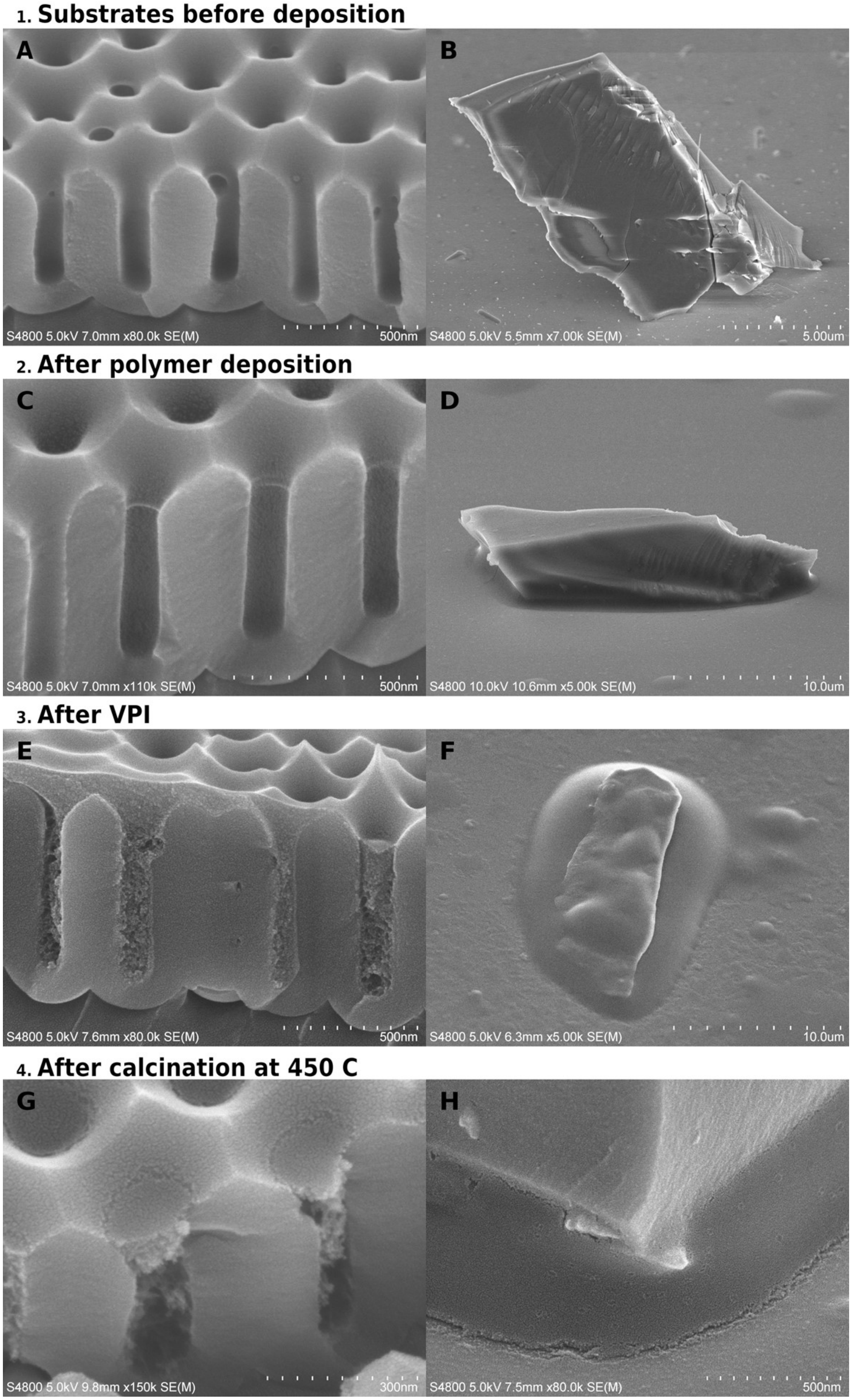

Figure S2: Tilted-view SEM images of AAO and glass substrates during different stages of the workflow. First row: empty pores and no filling below particles. Second row: Polymer has been deposited in the pores and below the particles. Third row: Polymer has been turned into a swollen hybrid material by VPI. Fourth row: the hybrid material was turned into inorganic depositions by calcination. The cross-sections were prepared by bending the surface right before sputtering and SEM imaging.

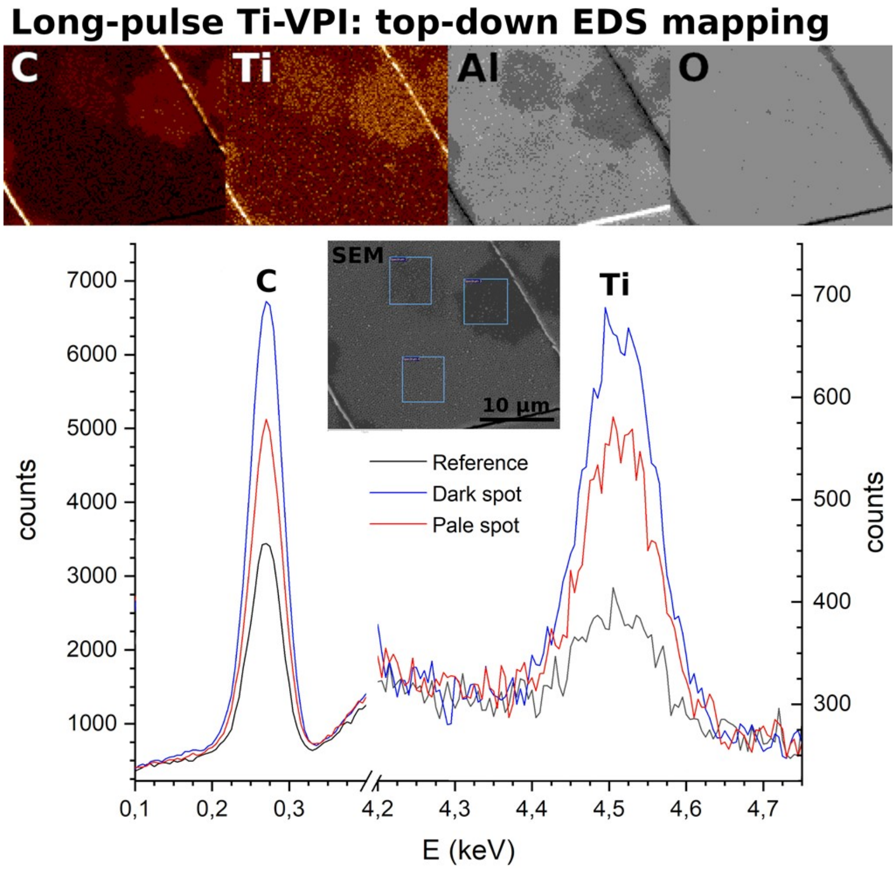

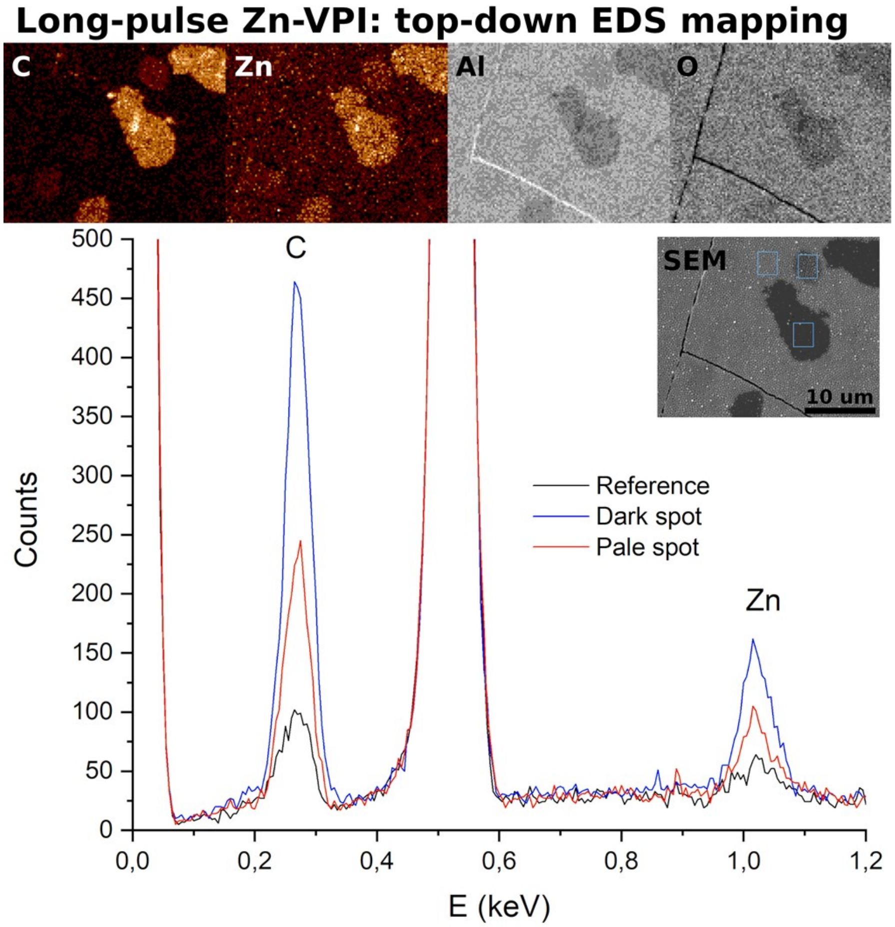

Figure S3. EDS elemental maps of long-pulse VPI experiments on AAO in top-down view. Dark and pale spots refer to areas where the polymer has stayed inside the pores (pale) or slightly overgrown the pore mouths (dark). The analyzed areas are marked in the SEM images with squares. Elemental maps and SEM images are as insets.

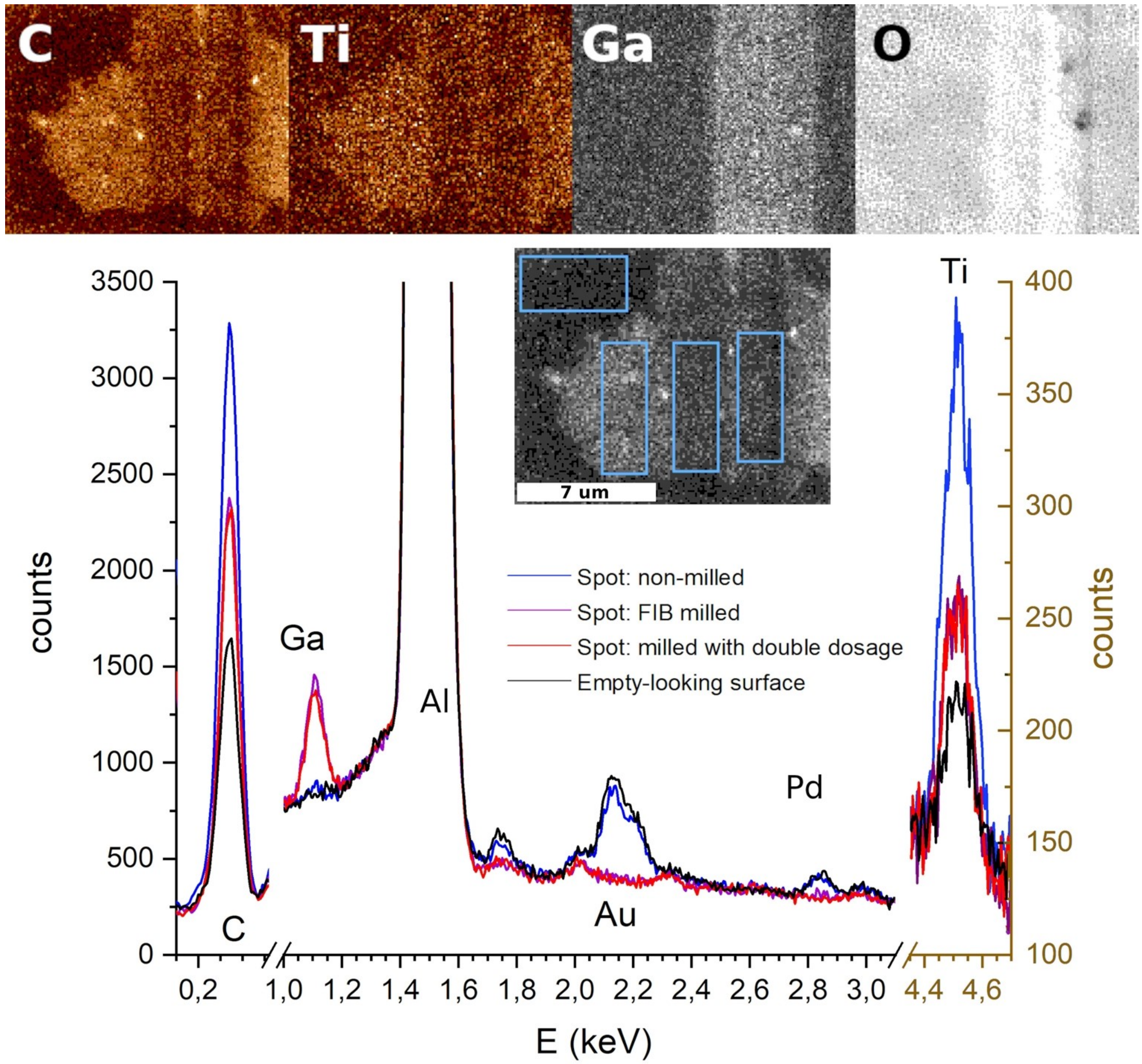

Figure S4. EDS elemental mapping of long-pulse TiO_x-VPI on AAO samples after focused ion milling with two dosages. Elemental maps and SEM image with analyzed areas marked as insets.

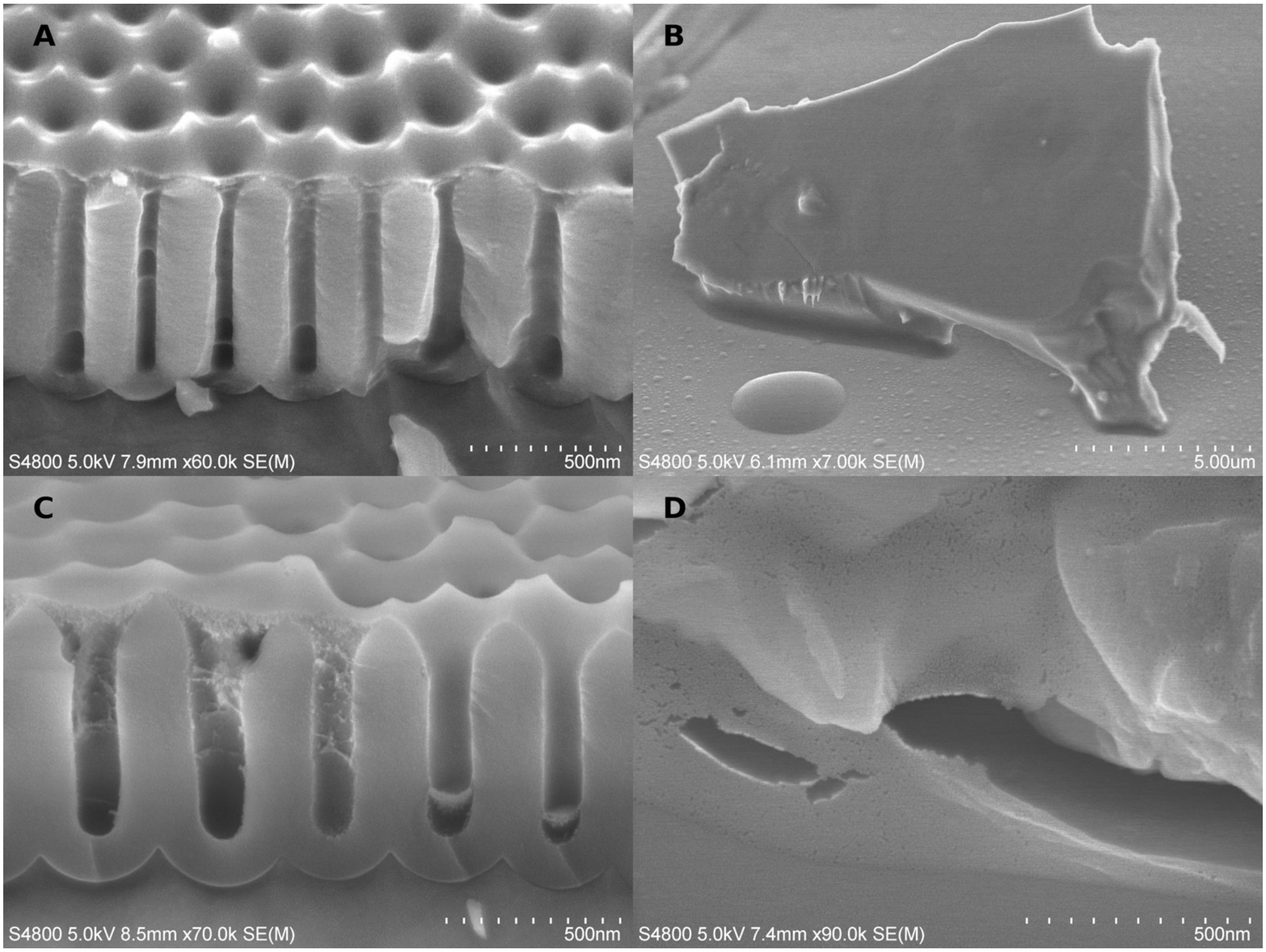

Figure S5. SEM images of short-pulse AlO_x-VPI samples with infiltration temperature of 60 °C. Hybrid material on A) AAO and B) glass. C) Slightly overfilled and two underfilled pores on AAO and D) a cracked thin film on a glass sample after calcination at 450 °C.

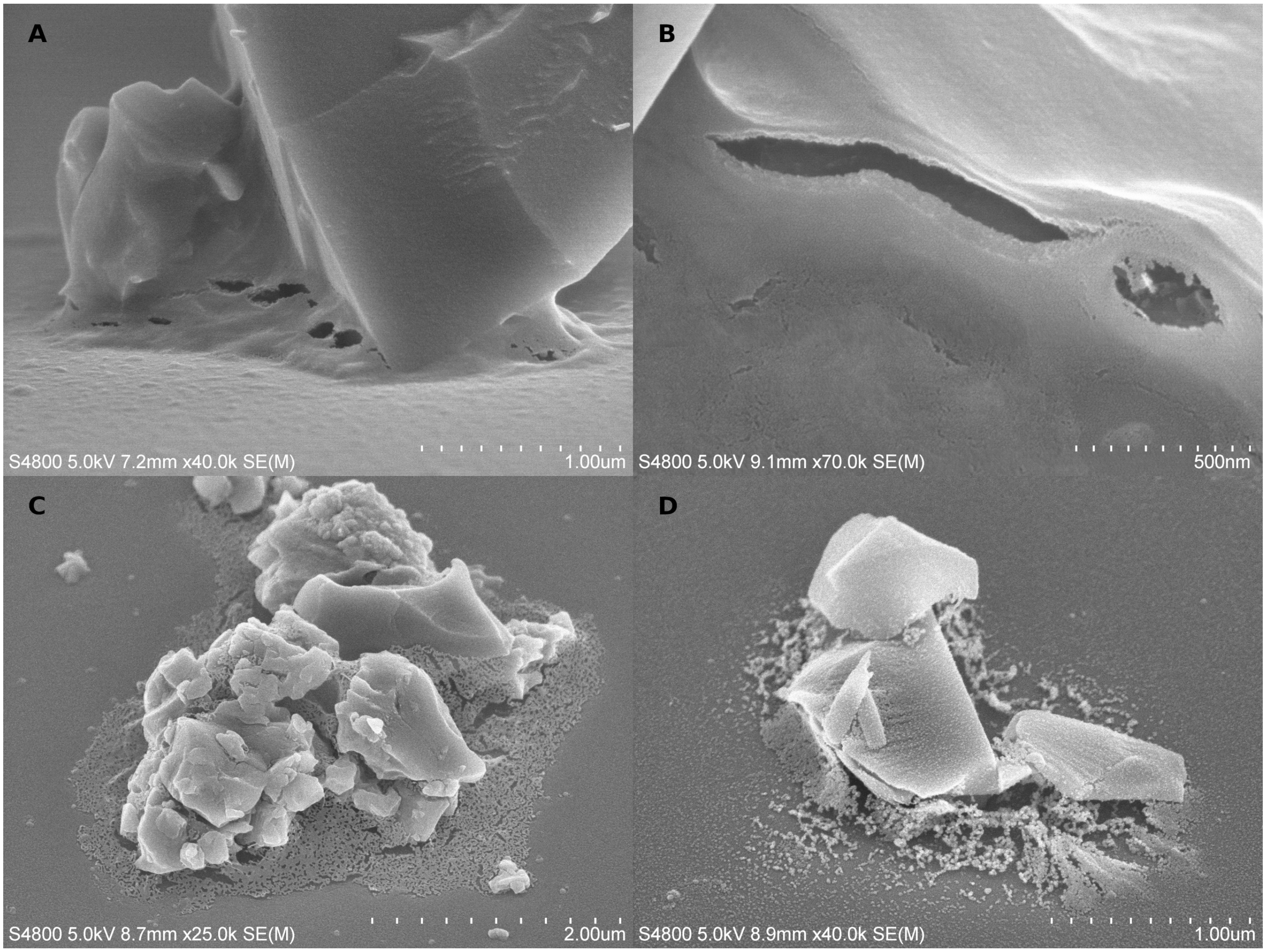

Figure S6. Long-pulse-VPI experiments on the glass substrate with O_2 present in the atmosphere during CT-piCVD. A, B) alumina VPI, when the polymer was deposited with 20% O_2 in the carrier gas, C) titania with 4% O_2 during CT-piCVD, and D) zinc oxide with 4% O_2 during CT-piCVD. The samples were calcined at 450 °C in air.
